# *Nocardia farcinica *lung infection in a patient with cystic fibrosis: a case report

**DOI:** 10.1186/1752-1947-4-84

**Published:** 2010-03-08

**Authors:** Fadi Bittar, Nathalie Stremler, Jean-Pierre Audié, Jean-Christophe Dubus, Jacques Sarles, Didier Raoult, Jean-Marc Rolain

**Affiliations:** 1 Unité de Recherche sur les Maladies Infectieuses et Tropicales Emergentes (URMITE), CNRS-IRD, UMR 6236, Faculté de Médecine et de Pharmacie, Université de la Méditerranée, Boulevard Jean Moulin, 13385 Marseille cedex 5, France; 2 Département des Maladies Respiratoires, Centre de Ressources et de Compétences pour la Mucoviscidose (CRCM), Hôpital Timone, Marseille, France; 3 Laboratoire de Bactériologie, Centre Hospitalier, Boulevard des Rayettes, 13698 Martigues CEDEX, France

## Abstract

**Introduction:**

Respiratory tract infections are the major causes of morbidity and mortality in patients with cystic fibrosis. *Nocardia *are rarely implicated in these infections and few reports of the involvement of this species are found in the literature.

**Case presentation:**

We describe a case of lung infection followed by chronic colonization of trimethoprim and sulfamethoxazole resistant *Nocardia farcinica *in a patient with cystic fibrosis. The chronic colonization of this uncommon bacterium in patients with cystic fibrosis was proved using a newly developed real-time polymerase chain reaction assay, which indicates that this bacterium, despite treatment, is difficult to eradicate.

**Conclusion:**

Our case report confirms that this organism can be recovered in persons with cystic fibrosis. Its eradication is necessary especially if the patient is to undergo lung transplantation.

## Introduction

Since the early description of cystic fibrosis (CF), pulmonary infection has been recognized as having the greatest role in morbidity and mortality leading to premature death in 90% of patients reported [1,2]. It has been demonstrated that accurate antimicrobial treatment is of great importance in avoiding the rapid destruction of the lung function of patients and in the spread of multidrug-resistant and/or highly virulent pathogens [1].

## Case presentation

In February 2007, a 15-year-old Caucasian French boy with CF (ΔF508, M1V heterozygote genotyping pattern) was admitted to the CF center in Marseille, France. He presented with fever (39°C), fatigue, haemoptysis, bronchial syndrome, and respiratory decompensation. His chest X-ray showed multiple bilateral bronchial thickening. He has had a history of persistent colonization of the respiratory tract since childhood with *Staphylococcus aureus *and *Haemophilus influenzae*. He also had a primary colonization with *Pseudomonas aeruginosa *at the age of 10 and with *Aspergillus fumigatus *and *Candida albicans *at the age of 13. Our patient received inhaled fluticasone (1500 μg/day) on several occasions in 2006.

**Figure 1:**
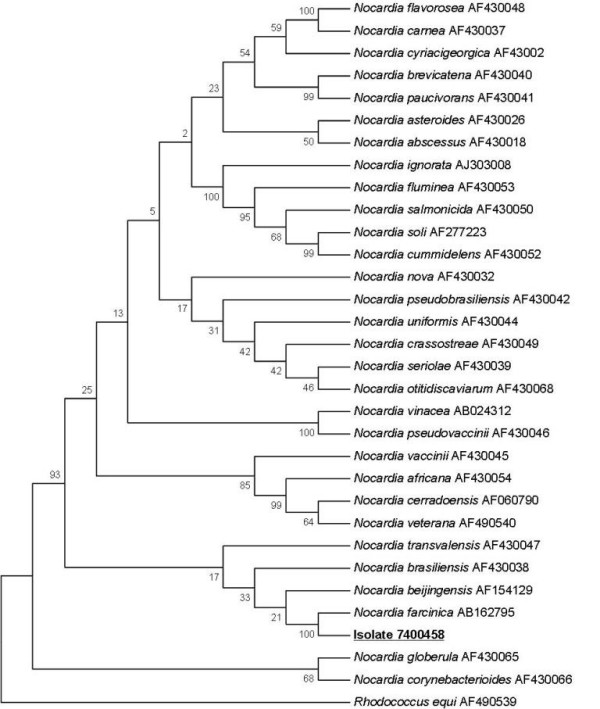
**Phylogenetic tree showing the position of *N. farcinica *(isolate 7400458, GenBank accession number **EU861514**) in bold and underlined within other *Nocardia species***. The tree was based on 16S rDNA comparison (1441 bp) using the neighbor-joining method from MEGA 4.0 software.

On admission, his laboratory tests showed a white blood cell count of 25.63 × 10^9^/L with 87% polymorphonuclear cells (PMNs). His C-reactive protein (CRP) was at 92 mg/L. A direct Gram-staining of his sputum taken at the time of his admission showed the presence of many polymorphonuclear leukocytes without squamous cells. The presence of Gram-positive branching filamentous bacilli and Gram-positive cocci was also noted. A standard culture and conventional identification of his sputum sample yielded 10^4^CFU/ml of *S. aureus*, 10^4^CFU/ml of *Nocardia *sp. that was isolated from Columbia colistin-nalidixic acid (CNA) agar (BioMérieux, Marcy l'Etoile, France) after three days of incubation. Accurate identification at species level was achieved after 16S rDNA amplification and sequencing [3] leading to the discovery of *N. farcinica *(GenBank accession number AB162795, 100%). The sequence of our isolate has been deposited in GenBank under accession number EU861514. The phylogenetic position of *N. farcinica *(strain 7400458) among closely related bacteria is presented in Figure 1. Using the disk diffusion method we found out that the isolate was resistant to rifampicin, erythromycin, gentamycin, doxycycline, trimethoprim/sulfamethoxazole and vancomycin. Meanwhile, we found it susceptible to amoxicillin, imipenem and ciprofloxacin.

Our patient subsequently received a three-week course of intravenous ciprofloxacin, amikacin, and trimethoprim-sulfamethoxazole. One month later, his leukocyte count decreased to 6.4 × 10^9^/L (50% PMNs), the culture was found negative, and our patient was declared clinically cured. During a follow-up examination after one year, *N. farcinica *was cultured four, six and nine months later and was not cultured at two and three months and one year later.

In December 2007, our patient was started on a six-month course of trimethoprim and sulfamethoxazole (160/800 mg, 1.5 tablets, b.i.d.) and nebulized amikacin (500 mg, b.i.d., one month/two during six months) in order to eradicate the bacterium. However, because of the difficulty in isolating *Nocardia species *in sputum samples, as well as the poor sensitivity of the culture method, we decided to investigate retrospectively all sputum samples available for our patient using a more sensitive assay (molecular detection). For this purpose we used a specific putative non-ribosomal peptide synthetase sequence [4,5] to develop an original real-time PCR assay with Taqman* probe. The primers designed for this RT PCR were as follows: NFF1 (5'-ACCGATCCGCCGTCAAATC-3') and NFR1 (5'-TCGGTCGTCCGGTGTGGA-3') that amplified a 147 bp of this gene with a probe of NFP1 (5'-CACATACCCCAACGCCAGCTGA-3'). The DNA from his sputa was extracted in a MagNa Pure LC instrument (Roche Diagnostics GmbH, Mannheim, Germany) according to the manufacturer's instruction.

**Figure 2:**
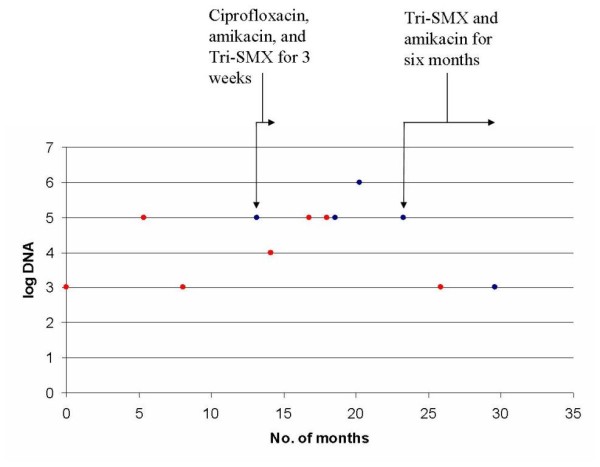
***Nocardia farcinica *DNA detection in different sputa recovered from the patient according the number of months and antibiotic treatment**. Month 0 = January, 2006. Blue point = *Nocardia farcinica *positive culture. Tri-SMX = trimethoprim-sulfamethoxazole.

Using our PCR assay, all investigated negative and positive *N. farcinica *culture sputa were positive in PCR even in sputa recovered one year before the first culture detection (Figure 2). Retrospectively, our patient's medical record was rechecked as his clinical condition, including cough, haemoptysis, purulent mucoid expectoration, and elevated CRP and leukocytes (> 80% PMNs) worsened at that stage.

## Discussion

*N. farcinica *is a Gram-positive branching filamentous bacillus causing many localized and disseminated infections in humans, including pulmonary and wound infections, brain abscesses, and bacteremia [6]. Immunocompromised hosts and patients undergoing immunosuppressive therapies are more commonly affected by this bacterium [6], but the isolation of *N. farcinica *from the respiratory tract of patients with CF is rare. Only three reports described the isolation of this bacillus in patients with CF: the case of a 31-year-old woman with deteriorated clinical parameters [7], the case of an 8-year-old boy who presented with fever and worsened chronic cough and who had previously received corticosteroid for allergic bronchopulmonary aspergillosis treatment [8], and the case of a 28-year-old woman with cough, dyspnea, fever, and hemoptysis and who had previously received corticosteroid [9]. Interestingly, our patient had a corticosteroid treatment history before the *Nocardia *isolation. Meanwhile, other *Nocardia *species have been reported in patients with CF, including *N. asteroides*, *N. asiatica*, *N. elegans*, and *N. transvalensis *[9,10].

*Nocardiae *are ubiquitous in the environment and can be found worldwide in fresh and salt water, in a variety of soil types, dust, decaying vegetation, and decaying fecal deposits from animals [6]. Dasgupta *et al. *described a patient who was an active gardener [7]. Interestingly, our patient also worked as a gardener, which led us to conclude that he might have acquired the infection in his job.

The first isolation of *N. farcinica *in our case was associated with clinical symptoms including fever, haemoptysis, bronchial syndrome, and multiple bilateral bronchial thickening and radiographic change. Our patient was then chronically colonized with this bacillus as proven by our new real-time PCR that appears more sensitive than culture (Figure 2). In the case reports of Isabel Barrio *et al.*, [9], six of 9 patients (67%) with *Nocardia *species isolations had clinical manifestations, while the *Nocardia *species were repeatedly isolated 3 patients. After a mean follow-up at 48 ± 33 months, however, the cultures were negative for all of the patients studied.

*N. farcinica *infections are potentially life-threatening [6,11]. The complete genome sequence of a clinical isolate (*N. farcinica *IFM 10152) revealed the presence of many candidate genes for virulence and antibiotic resistance [5]. After conducting a genomic analysis, the authors suggested that this bacterium can survive not only in soil environment but also in animal tissues, thus resulting in human disease [5]. It is now known *N. farcinica *is a multidrug-resistant bacterium that is susceptible mainly to amikacin, imipenem, trimethoprim/sulfamethoxazole, and ciprofloxacin [6]. Isolates from our patient were resistant to trimethoprim/sulfamethoxazole.

Cases involving *N. farcinica *that is resistant to trimethoprim/sulfamethoxazole have been described previously [5,12]. Chronic lung colonization in our patient, which was demonstrated by his positive PCR assay, indicates that antibiotic treatment does efficiently and completely eradicate the bacterium. In two recent case reports, the authors indicate that the need for *Nocardia *treatment should be evaluated on an individual basis and in the context of a clinical picture [9,10]. We believe that *Nocardia *eradication is needed in patients with CF, especially if they are due to undergo lung transplantation. The reason for this is that the immunosuppressive regimen during the pre- and/or post-operative period is a well-established risk factor for serious cases of nocardiosis and disseminated disease that leads to high rate of mortality [13].

The impact of the presence of multiresistant pathogens on the survival of patients with CF after lung transplantation remains controversial [14,15]. In the case of nocardiosis in patients with CF, no recommendations or data appear in the literature.

## Conclusion

We reported a case of lung infection followed by chronic colonization of trimethoprim-sulfamethoxazole resistant *N. farcinica *in a patient with CF. Our case report confirms that this infrequent organism can be recovered in such a population. We think that this unusual bacterium could be the cause of major clinical concern for patients with CF when immunosuppressive treatment is needed in cases requiring lung transplantation.

## Abbreviations

CF: cystic fibrosis; CNA: Columbia colistin-nalidixic acid; CRP: C-reactive protein; PMNs: polymorphonuclear cells.

## Competing interests

The authors declare that they have no competing interests.

## Authors' contributions

FB collected the data and drafted the manuscript. NS, JPA, JCD and JS took care of our patient during his hospitalization. DR and JMR participated in the design and critical revision of the study and helped draft the manuscript. All authors read and approved the final manuscript.

## Consent

Written informed consent was obtained from the patient for publication of this case report and any accompanying images. A copy of the written consent is available for review by the Editor-in-Chief of this journal.
